# Duplications and losses in gene families of rust pathogens highlight putative effectors

**DOI:** 10.3389/fpls.2014.00299

**Published:** 2014-06-26

**Authors:** Amanda L. Pendleton, Katherine E. Smith, Nicolas Feau, Francis M. Martin, Igor V. Grigoriev, Richard Hamelin, C. Dana Nelson, J. Gordon Burleigh, John M. Davis

**Affiliations:** ^1^Plant Molecular and Cellular Biology Program, University of FloridaGainesville, FL, USA; ^2^Southern Research Station, Southern Institute of Forest Genetics, USDA Forest ServiceSaucier, MS, USA; ^3^Department of Forest Sciences, University of British ColumbiaVancouver, BC, Canada; ^4^Interactions Arbres/Microorganismes, Laboratoire d'Excellence ARBRE, INRA-Nancy, UMR Institut National de la Recherche Agronomique – Université de LorraineChampenoux, France; ^5^US Department of Energy, Joint Genome InstituteWalnut Creek, CA, USA; ^6^Biology Department, University of FloridaGainesville, FL, USA; ^7^Genetics Institute, University of FloridaGainesville, FL, USA; ^8^School of Forest Resources and Conservation, University of FloridaGainesville, FL, USA

**Keywords:** effectors, rust pathogens, secretome, genome evolution, comparative genomics

## Abstract

Rust fungi are a group of fungal pathogens that cause some of the world's most destructive diseases of trees and crops. A shared characteristic among rust fungi is obligate biotrophy, the inability to complete a lifecycle without a host. This dependence on a host species likely affects patterns of gene expansion, contraction, and innovation within rust pathogen genomes. The establishment of disease by biotrophic pathogens is reliant upon effector proteins that are encoded in the fungal genome and secreted from the pathogen into the host's cell apoplast or within the cells. This study uses a comparative genomic approach to elucidate putative effectors and determine their evolutionary histories. We used OrthoMCL to identify nearly 20,000 gene families in proteomes of 16 diverse fungal species, which include 15 basidiomycetes and one ascomycete. We inferred patterns of duplication and loss for each gene family and identified families with distinctive patterns of expansion/contraction associated with the evolution of rust fungal genomes. To recognize potential contributors for the unique features of rust pathogens, we identified families harboring secreted proteins that: (i) arose or expanded in rust pathogens relative to other fungi, or (ii) contracted or were lost in rust fungal genomes. While the origin of rust fungi appears to be associated with considerable gene loss, there are many gene duplications associated with each sampled rust fungal genome. We also highlight two putative effector gene families that have expanded in *Cqf* that we hypothesize have roles in pathogenicity.

## Introduction

Rust fungi are plant infecting filamentous fungi in the order Pucciniales (Basidiomycota) that are unified by obligate biotrophy (Voegele and Mendgen, 2011). This form of pathogenicity requires a live host to establish a parasitic relationship. This is accomplished through the establishment of a molecularly intimate interaction at the host-pathogen interface characterized by the secretion of an arsenal of proteins from the pathogen that suppress host defense mechanisms and promote the acquisition of essential nutrients by the pathogen (Dodds et al., 2009; Stergiopoulos and de Wit, 2009). Such proteins, termed effectors, are thought to establish and maintain a compatible interaction between the pathogen and host. The processes that drive evolution of effector diversity are of great interest because pathogen's effector genes and host resistance genes are the interacting “gene-for-gene” pairs that drive coevolution in these pathosystems (Jones and Dangl, 2006; Stergiopoulos and de Wit, 2009).

Secreted proteins can be identified from whole genome sequences through the utilization of bioinformatic tools to isolate proteins with N-terminal secretion signals. Bioinformatic pipelines can then be used to narrow predicted secreted protein sets to putative effectors. These proteins contain features of known effectors such as elevated cysteine content (greater than 2%), that would enable the formation of stabilizing disulfide bridges (Stergiopoulos and de Wit, 2009), and protein domains associated with pathogenicity. Length is a criteria used to identify small secreted proteins (SSPs) from within putative effector protein sets, as SSPs are effector-like proteins with lengths less than 300 amino acids. Sequence comparisons alone do not provide a reliable means to identify putative effectors since some known effectors are lineage-specific while others are conserved across taxa (Rep, 2005; Saunders et al., 2012; Giraldo and Valent, 2013). Candidate effectors, a further distinction, are putative effectors that have additional support for roles in pathogenicity (i.e., induced transcription or elevated expression *in planta*). Genetic evidence for functional redundancy of effectors, presumably due to multigene families of effector proteins, whose members share similar functions, has been reported in several pathogens (Kamper et al., 2006; Rafiqi et al., 2012; Saitoh et al., 2012; Giraldo and Valent, 2013). This suggests it would be useful to characterize families of proteins with effector-like characteristics so as to identify families that have expanded during evolution in association with the acquisition of pathogenic life history characteristics. Examining the evolutionary history of protein families across a set of diverse fungal taxa should help identify lineage-specific, putative effector protein families, families that may have evolved similar functions in more distantly related taxa, and families that may exhibit functional redundancy.

*Cronartium quercuum* f. sp. *fusiforme* (*Cqf*) is a rust pathogen that has a complex life cycle with five spore types and exhibits alternation between two hosts, oak (*Quercus* spp.) and southern pines (*Pinus* spp.). The fungus incites fusiform rust disease on southern pines, leading to significant economic losses to the forest products industry. The impact of the disease on pine production has motivated extensive research on the genetic interaction between *Cqf* and pine. The objective of this study is to identify putative effector gene families in the *Cqf* genome through comparative genomic analyses between *Cqf* and 15 other fungal taxa, including two other rust pathogens. We have identified families that have expanded in *Cqf* that we hypothesize are involved in conditioning stem gall phenotypes observed on the pine host. Our analyses provide a more thorough perspective on *Cqf* and rust pathogen evolution and also highlight the evolutionary patterns of putative effector families that *Cqf* employs to establish disease on two taxonomically diverse host species.

## Materials and methods

### Gene family construction

Complete proteomes were downloaded from the public databases of the National Center for Biotechnology Information (www.ncbi.nlm.nih.gov/genome), U.S. Department of Energy's Joint Genome Institute (jgi.doe.gov/fungi), and the Broad Institute (www.broadinstitute.org). Sixteen proteomes were obtained: (Basidiomycota) *Cronartium quercuum* f.sp. *fusiforme* G11 version 1.0 (*Cqf*; unpublished, jgi.doe.gov/Cronartium), *Melamspora larici-populina* version 1.0 (*Mlp*; Duplessis et al., 2011a,b), *Puccinia graminis* f.sp. *tritici* CRL 75-36-700-3 race SCCL (*Pgt*; Duplessis et al., 2011a,b), *Mixia osmundae* IAM 14324 version 1.0 (*Mos*; Toome et al., 2014), *Sporobolomyces roseus* version 1.0 (*Sro*; with permission; jgi.doe.gov/fungi), *Rhodotorula graminis* strain WP1 version 1.1 (*Rgr*; with permission; jgi.doe.gov/fungi), *Ustilago maydis* strain 521 (*Uma*; Kamper et al., 2006), *Malasezzia globosa* CBS 7966 (*Mgl*; Xu et al., 2007), *Pisolithus tinctorius* Marx 270 version 1.0 (Pti; with permission; jgi.doe.gov/fungi), *Phanerochaete chrysosporium* version 2.0 (*Pch*; Martinez et al., 2004), *Heterobasidion irregulare* version 2.0 (*Hir*; Olson et al., 2012), *Serpula lacrymans* S7.3 version 2.0 (*Sla*; Eastwood et al., 2011), *Agaricus bisporus* var. *bisporus* H97 version 2.0 (*Abi*; Morin et al., 2012), *Laccaria bicolor* version 2.0 (*Lbi*; Martin et al., 2008), *Amanita muscaria* Koide version 1.0 (*Amu*; with permission; jgi.doe.gov/fungi), and (Ascomycota) *Saccharomyces cerevisiae* S288C (*Sce*; Goffeau et al., 1996), for a total of 200,313 proteins. Gene families were delineated by OrthoMCL v.5.0 software (Li et al., 2003) using default parameters (minimum *e*-value of 1e-05, minimum similarity of 50%).

### Secretome prediction

The collective set of secreted proteins, or the secretome, of *Cqf* was identified bioinformatically. Annotation of a secreted protein is determined by signal peptide (SignalP 3.0 and 4.0; Bendtsen et al., 2004; Petersen et al., 2011), protein localization (TargetP 1.1; Emanuelsson et al., 2000), and transmembrane domain (TMHMM 2.0; Krogh et al., 2001) bioinformatics prediction software (Feau et al. *in prep*.). Proteins predicted by TargetP 1.1 to be targeted for the mitochondrion (with RC values between 1 and 3) were discarded and residual proteins are submitted to TMHMM 2.0. If no TM-domain is identified in the protein, or a TM-domain is predicted in the N-terminal region of the protein (i.e., in the first 70 amino acids), the protein is re-oriented toward SignalP 4.0; in any other case, the protein is discarded. SignalP 4.0 either implements the SignalP-TM network to discriminate between a true signal peptide and an N-terminal trans-membrane region or the SignalP-noTM network if the program does not identify a TM-like domain in the N-terminal region of the protein. In this last case (i.e., if the the SignalP-noTM network is implemented by SignalP 4.0), the protein is re-oriented toward SignalP 3.0 and a signal peptide prediction is positive if either both NN and HMM converged in a positive result or if NN D-score returns a positive result with a D-score ≥ 0.5.

### Estimation of gene trees

The protein sequences from each gene family were aligned using MUSCLE (Edgar, 2004). We assembled a collection of amino acid alignments from gene families with at least four sequences. For each of the gene family alignments, we performed a maximum likelihood (ML) search to find the optimal topology using RAxML v.7.2.8 with the PROTCATJTT model (Stamatakis et al., 2005). Gene tree estimates often contain much error and can be improved with knowledge of the underlying species tree (e.g., Rasmussen and Kellis, 2011). We constructed a species tree from a phylogenetic matrix of 2404 single copy genes with sequences from at least eight fungal taxa. We performed a ML search using RAxML v.7.2.8 with the PROTCATJTT model on the concatenated single gene matrix to estimate the species tree. For each of the gene trees, we used TreeFix version 1.1.8 (Wu et al., 2013) to improve on the ML topology given the species tree. TreeFix searches for a statistically equivalent rooted gene tree topology that minimizes the number of duplications and losses implied by the species tree. For 10 of the gene families, the TreeFix runs did not complete in 1 week. For these gene trees, we rooted the ML tree with a root that minimizes the number of implied duplications and losses using the program OptRoot (www.wehe.us). For all of the gene trees output from TreeFix or OptRoot, the locations of the implied duplications and losses were mapped on the species tree using URec version 1.02 (Gorecki and Tiuryn, 2007).

### Functional annotation of proteins

Functional annotations were obtained from the Joint Genome Institute's (JGI) Mycocosm (jgi.doe.gov/fungi; Grigoriev et al., 2014) for the 16 organisms included in the phylogenetic and gene family analyses. Protein domains were identified using the online InterPro interface (http://www.ebi.ac.uk/interpro/; Hunter et al., 2012). Transmembrane domain regions were identified in amino acid sequences of proteins using TMpred (www.ch.embnet.org/software/TMPRED_form.html; Hofmann, 1993). Glycosylphosphatidylinositol (GPI) anchor sites were predicted using big-PI Predictor (http://mendel.imp.ac.at/gpi/gpi_server.html; Eisenhaber et al., 1999).

## Results

### Gene family analysis

**Figure 1:**
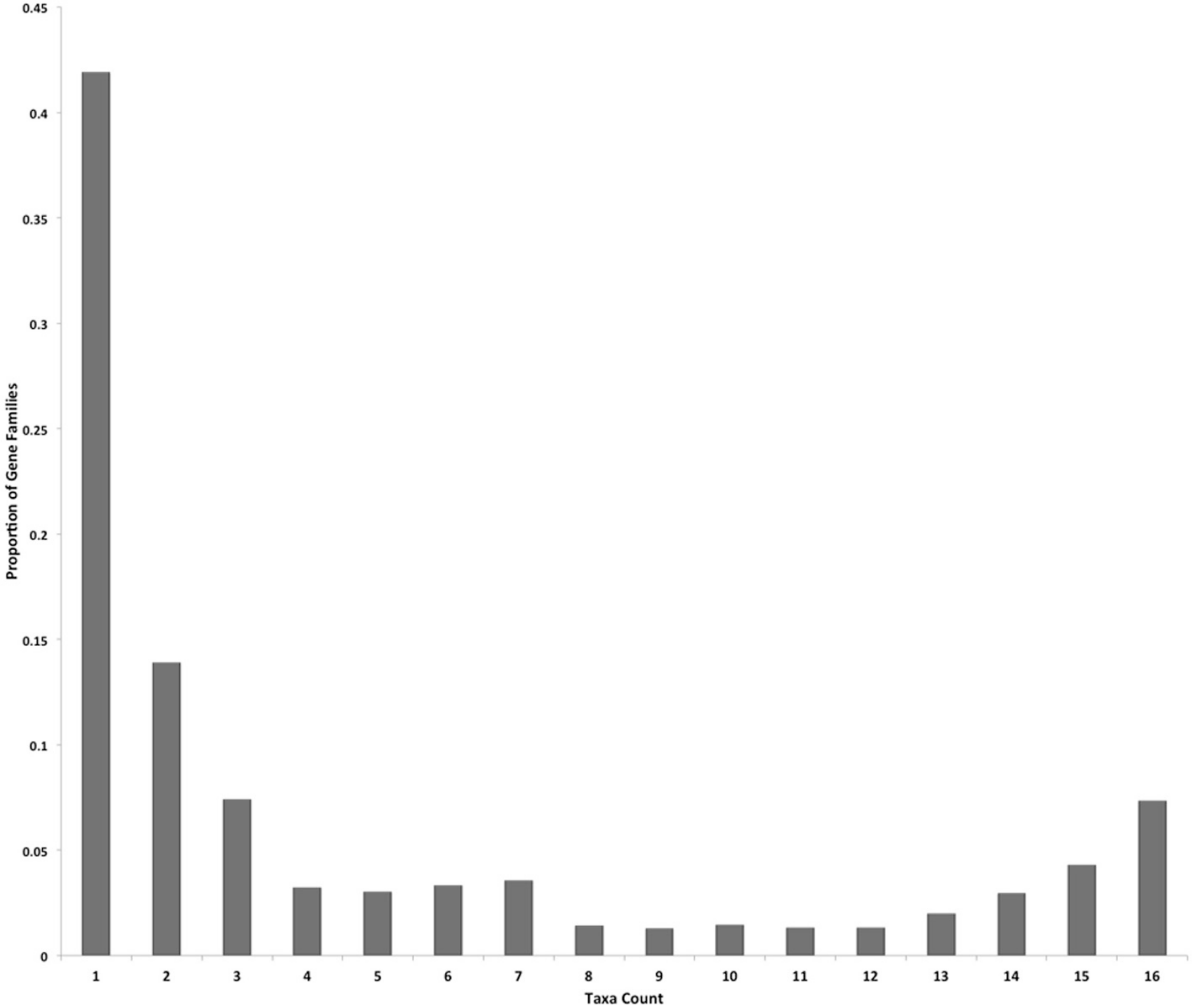
**Gene families are predominantly species-specific in the sampled taxa.** The proportions of gene families with proteins encoded in one through 16 fungal taxa genomes (taxa count) are displayed for the 19,489 OrthoMCL gene families.

The OrthoMCL analysis of the proteomes identified 19,489 gene families that contained 152,964 proteins. This protein count was ~76% (152,964/200,313) of the total proteins input into OrthoMCL analysis. Protein counts per gene family ranged from 2 (minimum size for a gene family) to 343 proteins, and the average family size was 7.8 proteins. Approximately 42% of the gene families had proteins encoded from only a single taxon, and families with proteins encoded in two or three taxa were the next most abundant families (Figure 1). Relatively few families contained proteins detected in 4–14 taxa, but more families contained proteins detected in 15 or 16 taxa (~12% of all families; 2,277/19,489). The families broadly conserved across all 16 sampled taxa are likely to contain core essential fungal proteins. The remaining ~24% of input proteins that did not group into families are considered true singletons, as they lack homologs within their own proteome or in the other taxa.

**Figure 2:**
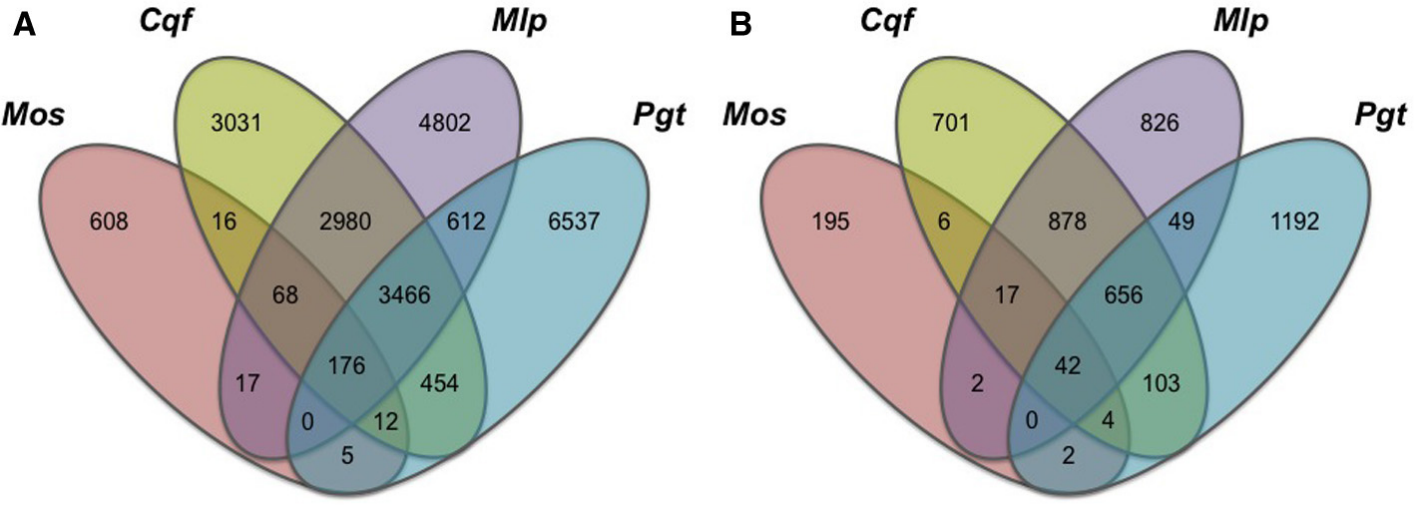
**Conserved proteins and families within only four Pucciniomycete pathogen genomes are mostly species-specific.** Gene family (OrthoMCL) conservation within Pucciniomycete pathogens; *Mixia osmundae* (Mos), *Cronartium quercuum* f.sp. *fusiforme* (Cqf), *Melampsora larici-populina* (Mlp), *and Puccinia graminis* f.sp. *tritici* (Pgt). The values indicate the total number of **(A)** gene models or **(B)** gene families conserved in only these four species and absent in the remaining 12 fungal taxa included in the OrthoMCL analysis.

To highlight gene families specific to the rust pathogen lineage, we compared gene family conservation between four pathogen genomes belonging to the subphylum Pucciniomycotina, which include three rust pathogens (*Cqf*, *Mlp*, and *Pgt*; Pucciniales) and a non-rust fern pathogen, *Mixia osmundae* (*Mos*; Mixiales). We selected the 4673 gene families containing proteins from at least one of these four pathogens (and no proteins from other sampled taxa) from the complete OrthoMCL family dataset. These families contained 22,784 proteins and exhibited varying patterns of conservation across the four taxa (Figure 2A). Most prominently, 14,978 of the 22,784 proteins (65.7%) were encoded in only one of the four pathogen genomes, illustrating high levels of species specificity (Figure 2A). Fewer proteins were shared between two or more rust fungi in this subset of families (7512/22,784 or 33.0%) (Figure 2A). Of the 19,485 families determined by OrthoMCL, 656 families (or 3.4%; Figure 2B) consisted of gene models found only in the three rust pathogen genomes, where each of the three rust pathogens had a representative gene model in the family. A total of 3466 proteins (Figure 2A) were ascribed to these rust pathogen-specific families. The largest family contained 249 proteins, and the smallest had 3 proteins. These 656 families represent the “core” rust pathogen protein set. Of the sampled genomes, the two pathogens with the most uniquely shared families are *Cqf* and *Mlp*, which have 878 conserved families.

### Identifying putative effectors

We identified gene families encoding putative effectors in the *Cqf* genome. To highlight putative effectors, the predicted secretome (predicted secreted proteins; see Methods) was analyzed for cysteine content and family-level conservation. The *Cqf* secretome harbors 666 SSPs, which are secreted proteins with fewer than 300 amino acids (aa). The range in protein lengths within the secretome was 51–1716 aa with a median length of 249 aa.

Analysis of *Cqf* gene families elucidated the evolutionary histories of secreted putative effectors. To identify putative effector families within the *Cqf* genome, we selected gene families with at least two secreted proteins, as these families would then contain at least two paralogous putative effectors and the family would have therefore expanded in the *Cqf* genome. In total, 132 putative effector families were identified. Sixty-five of these families were conserved effector families, with proteins from two or more fungal taxa. These families had sequences from 6.94 taxa on average (Table 1) and represent potential effectors with functions that can occur in a wide range of hosts. Alternatively, 67 novel effector families were considered to be evolutionary innovations since the family members consisted of only *Cqf* proteins (Table 2). The average family size for conserved effector families (18.23 proteins) was significantly larger than *Cqf*-specific families (3.54 proteins; *t*-test, *p*-value < 0.001). However, there was no difference in the number of *Cqf* proteins per family in conserved (mean = 5.02 proteins) and *Cqf*-specific families (mean = 2.4 proteins). Families where all *Cqf* protein members are predicted to be secreted were found in both candidate effector family types and at proportions that were not significantly different from one another (conserved families = 40/65, *Cqf*-specific = 44/67; Tables 1, 2). Evidence for potential sub- and/or neofunctionalization was observed in 23 of the 67 (34.3%) *Cqf*-specific putative effector families, as only a subset of proteins within these families received secretion predictions, suggesting distinct biological roles among family members.

**Table 1:** **Conserved putative effector families have broad and narrow taxonomic distributions**.

**OrthoMCL group ID**	**Total proteins in family**	***Cqf* proteins in family**	**Proteins in *Cqf* secretome**	**Number of taxa represented in family**
**5485**	12	7*	7	3
2168	17	6	5	3
2725	16	13	5	3
1053	94	6	4	13
2731	16	5	4	5
5853	11	5	4	5
6604	9	5	4	2
1101	65	62	3	2
1281	33	3*	3	3
1293	32	5	3	10
1397	27	3*	3	12
1831	19	3*	3	6
2730	16	3*	3	10
5067	13	4	3	7
6199	10	3*	3	3
6608	9	3*	3	3
6599	9	3*	3	5
7036	8	4	3	2
9412	5	3*	3	2
10,437	4	3*	3	2
1014	170	69	2	10
1219	39	3	2	14
1382	28	2*	2	13
1410	27	3	2	15
1444	26	2*	2	14
1428	26	2*	2	10
1507	24	3	2	15
1546	23	2*	2	13
1541	23	2*	2	16
1593	22	2*	2	13
1703	21	2*	2	8
1898	19	2*	2	14
1829	19	2*	2	15
1957	18	2*	2	14
2180	17	3	2	9
2191	17	2*	2	10
2172	17	2*	2	16
4359	15	2*	2	13
3833	15	2*	2	12
3825	15	2*	2	11
4560	14	2*	2	10
5075	13	2*	2	7
5492	12	3	2	6
5799	12	2*	2	10
6367	10	3	2	6
6213	10	3	2	3
6206	10	2*	2	3
6601	9	7	2	2
6606	9	3	2	7
6629	9	2*	2	6
7040	8	2*	2	2
7590	7	2*	2	3
9416	5	4	2	2
9414	5	4	2	2
9843	5	2*	2	3
9462	5	2*	2	2
9446	5	2*	2	2
9431	5	2*	2	4
9424	5	2*	2	3
10,479	4	3	2	2
10,474	4	3	2	2
10,432	4	3	2	2
11,968	3	2*	2	2
11,958	3	2*	2	2
11,908	3	2*	2	2
Total	1185 proteins	326 proteins	162 proteins	-
Average	18.23 proteins per family	5.02 *Cqf* proteins per family	2.5 secreted proteins per family	6.94 taxa per family

Gene families with greater than two Cqf predicted secreted proteins are listed. Data is ranked by the number of Cqf secreted proteins. The total number of proteins in each family is provided as well as the number of proteins belonging to the Cqf secretome (i.e., predicted secreted proteins). Asterisks adjacent to total protein counts indicate families where all members are Cqf secretome members. If no asterisk is present, only a portion of the family received secretion predictions. Family 5485 (bold) will be detailed later in article.

**Table 2:** **Potential sub- and neo-functionalization within *Cqf-*specific putative effector families**.

**Gene family ID**	***Cqf* proteins in family**	**Proteins in *Cqf* secretome within family**
8514	6*	6
**9417**	5*	5
9892	5*	5
2663	17	4
6030	11	4
9436	5	4
9897	5	4
7037	8	3
9890	5	3
9891	5	3
10,467	4	3
11,111	4	3
11,876	3*	3
11,879	3*	3
11,934	3*	3
12,788	3*	3
12,794	3*	3
12,804	3*	3
12,812	3*	3
5202	13	2
7921	7	2
7928	7	2
8502	6	2
9880	5	2
10,433	4	2
10,463	4	2
11,112	4	2
11,928	3	2
11,944	3	2
12,777	3	2
12,803	3	2
12,807	3	2
12,814	3	2
14,526	2*	2
14,527	2*	2
14,537	2*	2
14,552	2*	2
14,554	2*	2
14,563	2*	2
14,570	2*	2
14,577	2*	2
14,589	2*	2
14,623	2*	2
15,977	2*	2
15,979	2*	2
15,980	2*	2
15,992	2*	2
16,012	2*	2
16,034	2*	2
16,036	2*	2
16,051	2*	2
16,052	2*	2
16,078	2*	2
16,079	2*	2
16,080	2*	2
16,081	2*	2
16,091	2*	2
16,101	2*	2
16,102	2*	2
16,106	2*	2
16,119	2*	2
16,123	2*	2
16,129	2*	2
16,146	2*	2
16,160	2*	2
16,163	2*	2
16,191	2*	2
Total	237 proteins	164 proteins
Average	3.54 *Cqf* proteins per family	2.4 secreted proteins per family

Cqf-specific gene families with greater than two predicted secreted proteins are listed. The total number of proteins in each Cqf-specific family is provided as well as the number of proteins belonging to the Cqf secretome (i.e., predicted secreted proteins) are indicated. Asterisks adjacent to total protein counts indicate families where all members are Cqf secretome members. If no asterisk is present, only a portion of the family received secretion predictions. Family 9417 (bold) will be detailed later in article.

### Gene gains and losses

**Figure 3:**
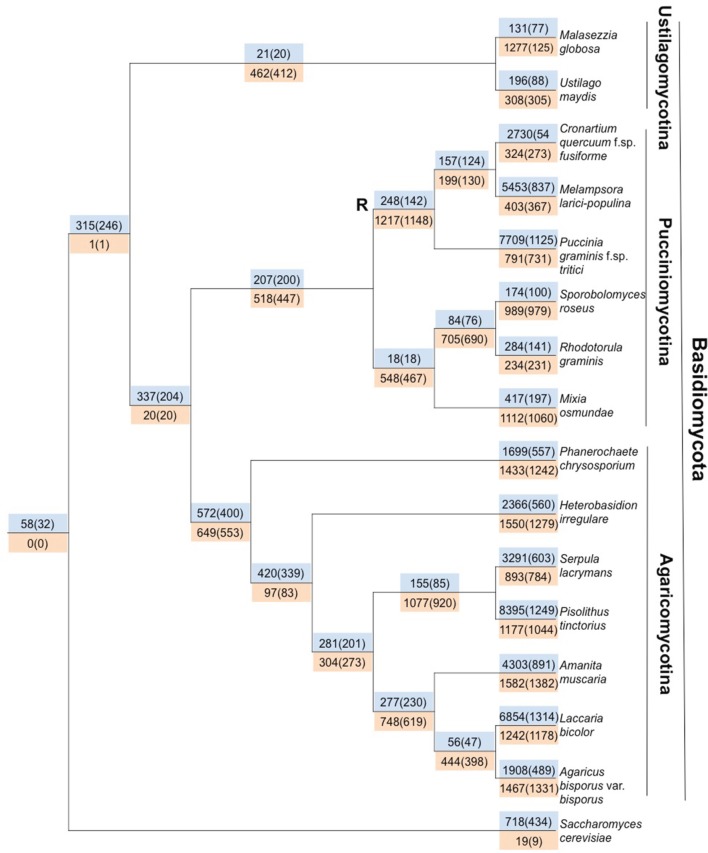
**Gene loss and gain in the Basidiomycota fungal lineage highlights shared loss in the rust pathogen lineage and high levels of species-specific gain.** Mapping putative gene duplications and losses across 16 fungal taxa. Values in blue are associated with gains/duplications, whereas orange indicates loss. Outside of parentheses are the number of gain or loss events that have occurred on the branch preceding a node, and within parentheses are the number of gene families associated with duplications or losses. The node denoted with R indicates the last common ancestor of the rust pathogens.

**Figure 4:**
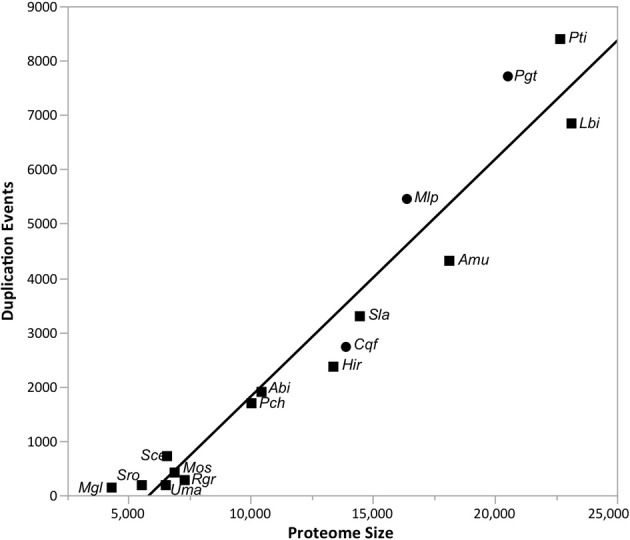
**The proteome size is positively correlated with the number of gene family duplications across taxa.** The linear relationship (*R*^2^ = 0.93) between the proteome size (i.e., protein count) and the number of species-specific duplications detected in the analyses from Figure 1 is depicted in this figure. Rust pathogens (*Cqf*, *Mlp*, and *Pgt*) are indicated with circles and non-rust pathogens with squares. The line of best fit (black) is indicated. Please reference Methods for species abbreviations.

**Figure 5:**
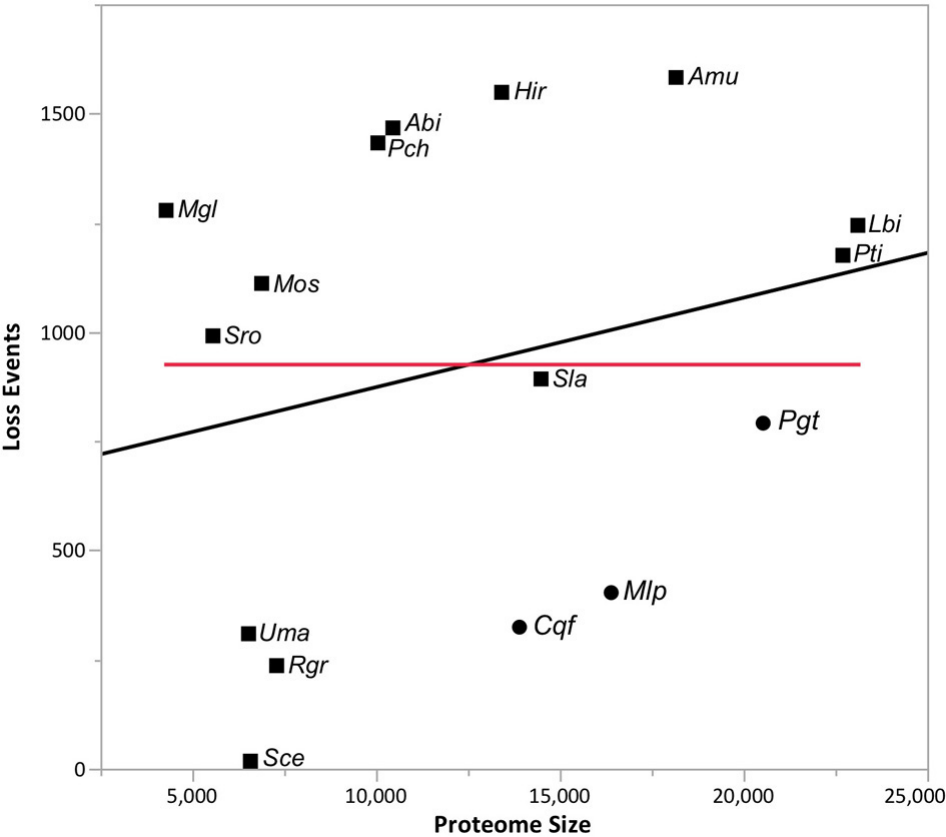
**Lack of relationship between proteome size and gene family losses across taxa.** The relationship (*R*^2^ = 0.06) between the proteome size (i.e., protein count) and the number of species-specific losses detected in the analyses from Figure 1 is depicted in this figure. The line of best fit (black) and fit mean (red) are also shown. Rust pathogens (*Cqf*, *Mlp*, and *Pgt*) are indicated with circles and non-rust pathogens with squares. Please reference Methods for species abbreviations.

Gene gain and loss was quantified across all 16 sampled fungal taxa. We mapped the gene trees from gene families with at least four proteins onto a species tree to determine the patterns of duplication and loss across the 16 fungal taxa. In total, we examined 10,371 gene trees containing 131,863 protein sequences. These gene trees implied a minimum of 49,539 duplications (i.e., gene family gains) and 21,789 losses (i.e., gene family contractions and/or entire family loss). Over 93.9% of the duplications and 67.9% of the losses are species-specific, occurring in a single lineage at the tips of the species tree (Figure 3). The number of species-specific duplications was positively correlated with the size of a taxon's proteome (*R*^2^ = 0.93), suggesting that gene duplication is a mechanism for proteomic expansion and diversification for the selected fungal taxa (Figure 4). There was no obvious relationship between proteome size and species-specific duplication with life history forms (i.e., symbiotic, pathogenic, or free-living) (Figure 4). Species-specific losses were not correlated with the proteome size, but the rust pathogen lineage exhibited fewer losses than other sampled taxa (Figure 5).

**Figure 6:**
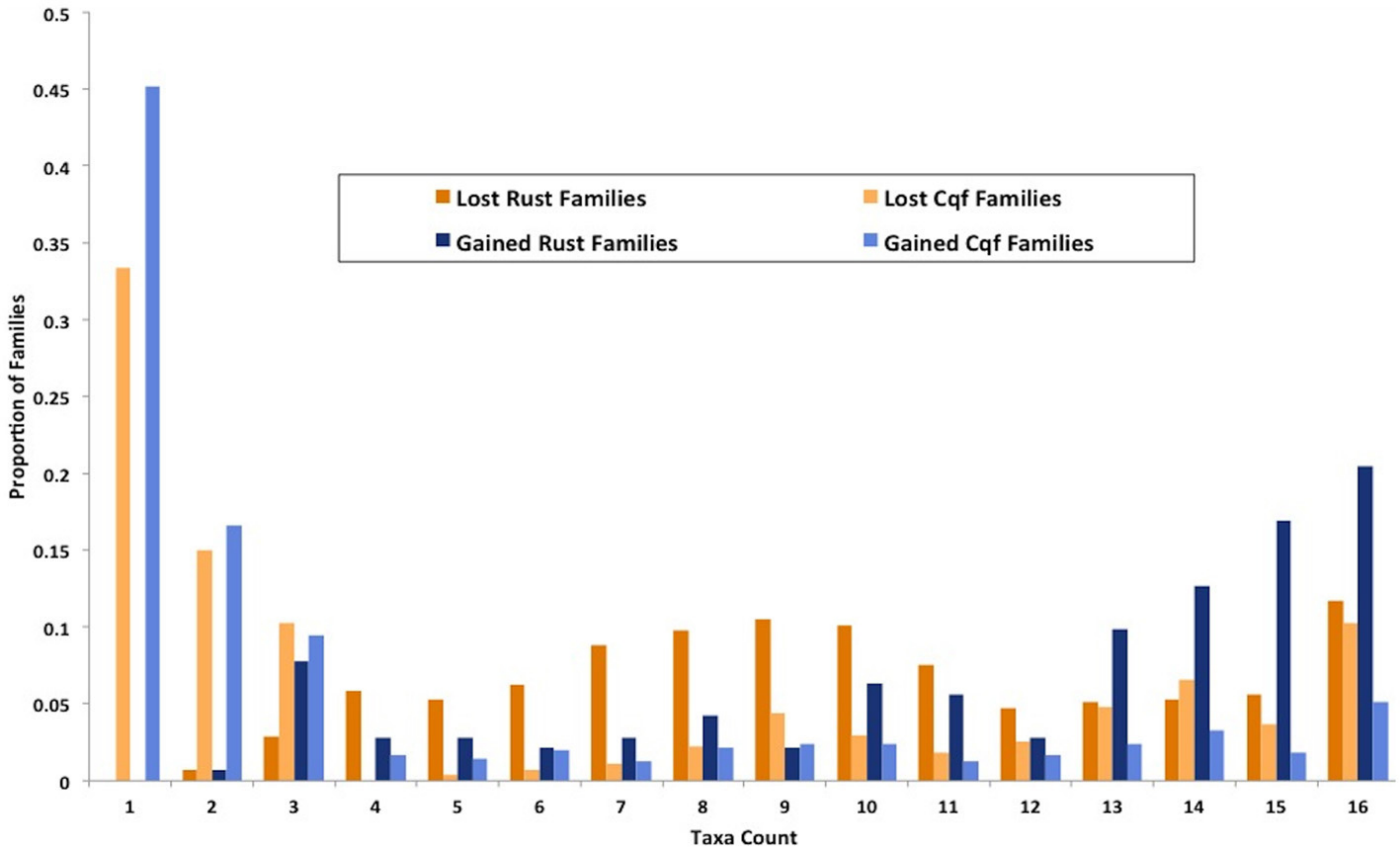
**Families gained and lost in rust pathogens and *Cqf* have varying levels of taxonomic conservation.** The proportion of gene families (y-axis) that contain protein members from 1 to 16 fungal taxa (x-axis) among those that have either expanded or contracted in the rust fungi or *Cqf*.

We identified genes that were gained and lost specifically in the rust pathogen clade. There were many gene losses (1217 events within 1148 families) associated origin of the rust pathogen clade within Pucciniomycotina (*Cqf*, *Mlp*, and *Pgt*) compared to the number of gains (248 events, 142 families) (site R in Figure 3). The number of taxa represented in these 1148 families range from 2 to 16 species, with the largest proportion of families (10.6%) having representatives from all 16 taxa in the analysis (Figure 6). Families lost genes at the origin of the rust fungi appear to occur in few of the sampled fungal lineages than those that had duplications in the rust fungi. Fifty percent of duplicated families contain proteins from 14 or more sampled taxa (Figure 6). Though the disproportionate level of gene losses prior to the common ancestor of rust pathogens is striking, each of the three rust fungal species shows evidence of high species-specific rates of duplication (Figure 3). In fact, 32.1% of all the duplications across the tree are specific to only one of the rust species (Figure 3).

The proteome of the *Cqf* rust pathogen is enriched for novel proteins whose expansion has presumably contributed to specialization in its pathosystem. Numerous species-specific duplications have occurred following within the *Cqf* lineage (2730 duplication events in 549 families; Figure 3). Of the 549 families that have undergone *Cqf*-specific duplications, 248 (or 45.17%) contain proteins not observed in any other analyzed fungal taxa. These 248 novel families comprise 14.5% of the annotated *Cqf* proteome (2017/13,903 proteins), highlighting the rapid expansion of novel, likely pathogenicity-related gene families. The vast majority (98.8%) of these novel families do not have BLASTp hits in the NCBI non-redundant database or have hits to unknown proteins (minimum *e*-value of 1e-10; Table 3) and 94.5% do not contain InterPro domains (unpublished, jgi.doe.gov/Cronartium; Hunter et al., 2012). Since the families that are unique to the *Cqf* lineage are largely uncharacterized, they likely follow the assumptions for putative pathogenicity factors or effectors. Nearly 12% (234/2,017) of proteins encoded in the 248 novel *Cqf* families are members of the predicted *Cqf* secretome. This is significantly greater than the ~8% of entire *Cqf* proteome that also belongs to the secretome (Chi-square = 25.418, *p*-value = 0.0001). The protein characteristics of these secreted proteins are effector-like, as the average cysteine content is 2.2% and the median protein length is 272 amino acids.

**Table 3:** ***Cqf*-specific duplicated gene families contain predominantly uncharacterized proteins**.

**Gene family annotation**	**Number of gene families (proteins)**
No hits	183 (1510)
Unknown protein	57 (430)
20S Proteasome subunit alpha 6	2 (9)
Zinc finger CCHC-type protein	1 (33)
HIV-1 retropepsin, polyprotein	1 (13)
Polysaccharide lyase family 4	1 (7)
Reverse transcriptase	1 (5)
MFS transporter, inorganic phosphate transporter	1 (5)
CFEM domain containing protein	1 (5)

Functional annotation of the 248 gene families duplicated only in Cqf by BLASTp against the non-redundant NCBI database (minimum e-values of 1e-10). A family was ascribed a function if more than two proteins in the family received the same top annotated BLASTp hit. The number of proteins within families is indicated in parentheses.

The families that were duplicated in the *Cqf* lineage and contain sequences from other taxa exhibit patterns of conservation that differ from the families duplicated or depleted in rust pathogens (site R, Figure 3). Instead, these *Cqf*-specific duplicated families (*n* = 549 families) are predominantly conserved in not only *Cqf*, but also 2-3 taxa (Figure 6).

### *Cqf* putative effector gene families—distribution and expansion

**Figure 7:**
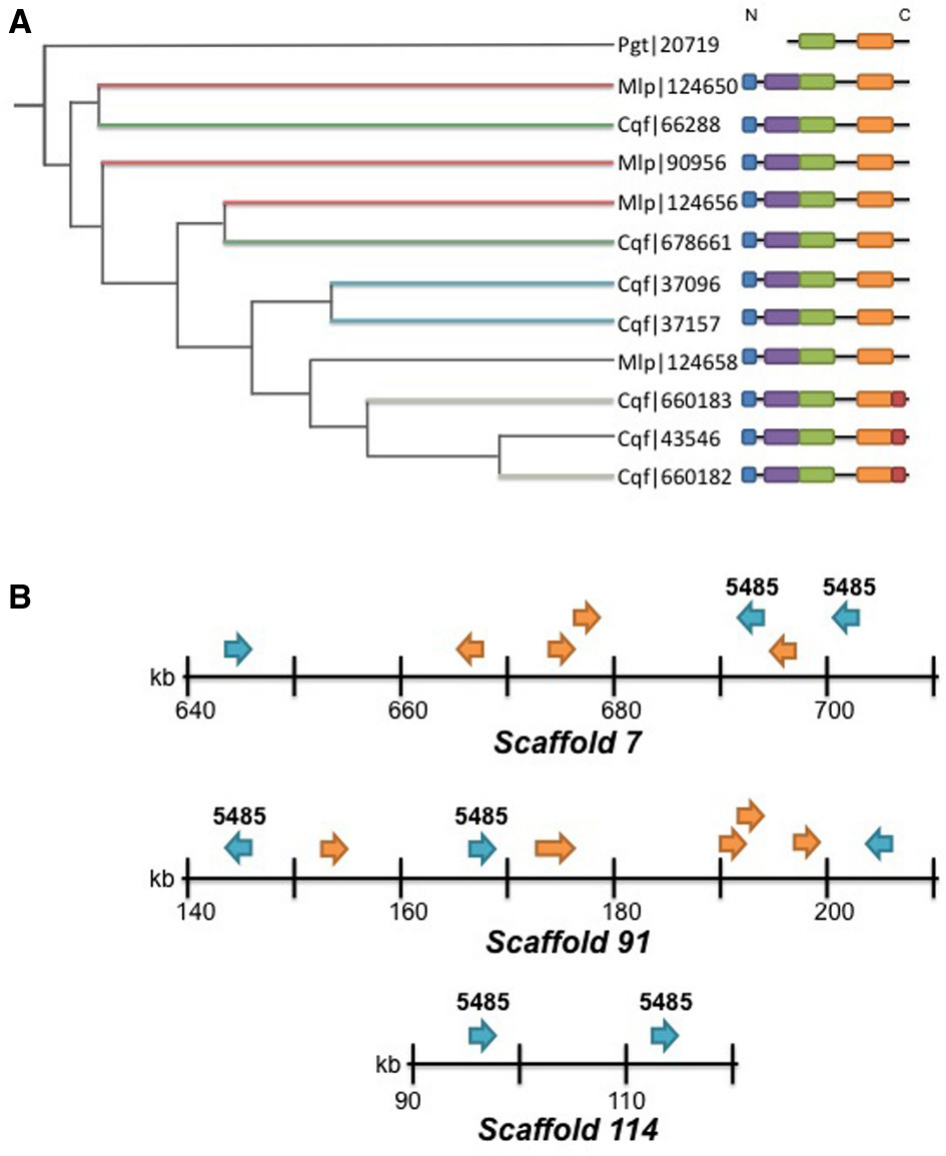
**Domain structure, phylogenetic relationships, and colocalization of copper oxidases. (A)** Phylogenetic relationship estimated with TreeFix (Wu et al., 2013) for the 12 members of Family 5485. Tree reconstructed with Tree of Life viewer (Letunic and Bork, 2011). See methods for additional details for gene tree estimation. Branch lengths are not informative for this tree. Domain architectures are indicated for each protein: N-terminal signal peptides per SignalP 4.0 (blue; Petersen et al., 2011); (MCu1—IPR01117) Multicopper oxidase Type 1 (green); (MCu2—IPR011706) Multicopper oxidase Type 2 (orange); (MCu3—IPR011707) Multicopper oxidase Type 3 (purple); and (CuBS—IPR002355) Multicopper oxidase, copper-binding site as determined by InterPro (red; Hunter et al., 2012). The colocalization of proteins on scaffolds is indicated by branches sharing the identical colors. Thin black branches do not colocalize. **(B)** Co-localization of proteins within MCO Family 5485 on scaffolds 7, 91, and 114. Family 5485 members are denoted with the ID above each gene model. Secretome members are blue arrows and non-secreted proteins are orange arrows. Gene orientation on the scaffold is indicated with arrows. Note: gene lengths are not to scale.

Family 5485 is the largest family represented in the predicted *Cqf* secretome. The family contains 12 orthologous proteins (7 *Cqf*, 4 *Mlp*, and 1 *Pgt* proteins). Eleven of the 12 proteins have predicted N-terminal signal peptides (SignalP 4.0; Petersen et al., 2011), and all seven members from *Cqf* are annotated as belonging to the *Cqf* secretome. Domain architecture and conservation data for Family 5485 proteins helps to predict their biological functions and putative roles in establishing infection. Additionally, 11 of the 12 proteins in this family contained three multicopper oxidase (MCO) domains and the remaining protein (Pgt_20719) contained two of the three domains (Figure 7A). The Interpro domains identified include: Cupredoxin domain (IPR008972), Multicopper Oxidase, Type 1 (IPR001117), and Multicopper Oxidase, Type 2 (IPR011706), and Multicopper Oxidase, Type 3 domain (IPR011707) (Figure 7A). A Copper-Binding Site domain (IPR002355) was identified in only three *Cqf* family members. These three proteins have a distinct phylogenetic history from other family members (Figure 7B). Generally, the phylogenetic relationships, as well as the genomic colocalization of the proteins in this family mirrors the domain architecture, providing insight into how these proteins evolved (Figure 7A). Several additional families of MCOs are present in the *Cqf* genome (i.e., Families 5853, 1542, and 1053), however, by definition, Family 5485 has a distinct evolutionary history from other families as evidenced by distinct family placement by OrthoMCL.

**Figure 8:**
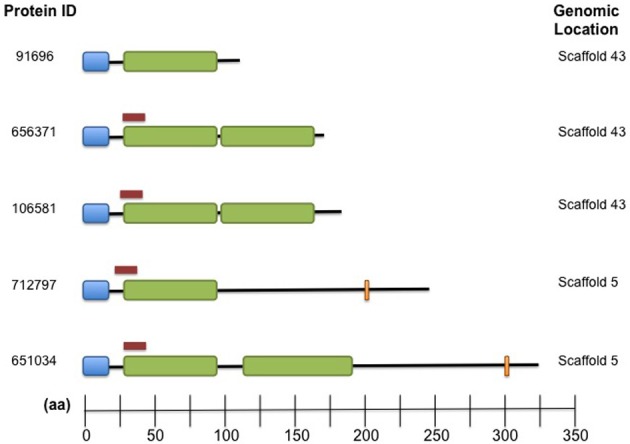
**Family 9417 proteins harbor both signal peptides and CFEM domains domains and colocalize in the *Cqf* genome.** Domain architecture of Family 9417 protein members where signal peptides (blue), CFEM Interpro domains (green), GPI anchor sites (orange), and predicted transmembrane domains (red) are indicated for each protein. Three proteins colocalize together on scaffold 43, the remaining on scaffold 5 (indicated on right).

Family 9417 is the third largest family in the *Cqf* secretome, with all five of its proteins predicted as secreted. This family contains putative effectors likely involved in the establishment of disease, as all members have signal peptides, short lengths (average 207 aa), and high cysteine content (average 6.5%). All five family members contain at least one fungal extracellular membrane (CFEM) domain (Interpro IPR008427). Five additional proteins encoded in the *Cqf* proteome contain CFEM domains. Two of the five do not belong to a gene family, and the remaining three proteins each were ascribed to different families containing orthologs from multiple fungal taxa, unlike *Cqf*-specific family 9417. Similar to Family 5485, proteins of Family 9417 also colocalize in the genome, as three members are located on scaffold 43 of the *Cqf* assembly and the remaining two proteins are adjacent to one another on scaffold 5 (Figure 8). Protein members of Family 9417 adhere to consensus domain structure and subcellular targeting of previously identified CFEM proteins. Online prediction algorithms detected transmembrane domain regions (Tmpred; Hofmann, 1993) and glycosylphosphatidylinositol (GPI) anchor sites (big-Pi Predictor; Nielsen et al., 1997) in a subset of the family proteins (Figure 8). All proteins, excluding Cqf91696, were predicted to have N-terminal transmembrane helices spanning amino acids 3-23 for both proteins. Two members, Cqf712797 and Cqf651034 had C-terminal GPI anchor sites at amino acids 223 and 302, respectively (*p*-values 1.25E-04 and 2.10E-04). Only Cqf91696 had no bioinformatic evidence of association with the fungal membrane.

## Discussion

This study provides the first detailed analysis of the secretome of the fusiform rust pathogen, *Cqf*, since the recent assembly and annotation of a draft reference genome (unpublished, jgi.doe.gov/Cronartium). Additional criteria used in isolating putative effectors from within the *Cqf* genome and its corresponding secretome, included proteins exhibiting rust pathogen-specific and *Cqf*-specific gene family membership. Following gene family constructions, we highlighted putative effectors with paralogs (within *Cqf*) or orthologs/paralogs (between *Cqf* and other taxa) within the *Cqf* secretome. Over half (51%) of proteins considered to be effector-like (small, cysteine-rich, secreted proteins) belong to gene families. This is comparable to results found in the hemibiotrophic pathogens *Phytophthora ramorum* and *P. sojae* where 77% of their secretomes are found in multigene families (Tyler et al., 2006). These findings demonstrate the value of an evolutionary perspective for highlighting families harboring putative *Cqf* effectors. Altogether, the large-scale comparative genomics analyses in this study help elucidate the unique patterns of evolution in a rust proteome and its associated secretome.

### Putative effector families

With the completion of the *Cqf* draft genome, it is important to identify proteins that may be involved in establishing disease, such as effectors, on oak and pine hosts. Based on the evolutionary forces presumed to act on effectors, in combination with a trio of rust pathogen genomes facilitating comparative analyses, we can now do experiments not previously feasible. We suggest this is a reasonable approach to identifying putative effectors that complements more conventional methods. Previous studies searched for effectors within other systems based on the presence of a signal peptide, cysteine richness, and short protein lengths (<300 aa) (Joly et al., 2010; Cantu et al., 2011; Duplessis et al., 2011b; Hacquard et al., 2012; Saunders et al., 2012). This study highlights the usefulness of comparative genomic analyses to examine the evolutionary history of each secretome member, and that this approach can also be complemented with structural characteristics of predicted secreted proteins. The rationale behind these comparisons is that effector families conserved in rust fungi and unique to *Cqf* are candidates for conditioning rust pathogen and *Cqf* infection strategies, respectively.

We observed species-specific proteomic gene family gains/duplications in the *Cqf* lineage, a subset of which represents putative effectors. The paralogous nature (i.e., multi-copy) of their protein family members indicates functional redundancy, which is consistent with other pathogenic fungi (Kamper et al., 2006; Saitoh et al., 2012). We have identified two lines of evidence that point toward neo- and sub-functionalization in *Cqf* putative effector families. First, differential subcellular localization predictions have been observed within putative effector families. In about 34% of *Cqf*-specific families, only a subset of proteins are secreted from the fungal cell, while remaining family members are not predicted for secretion, thus remaining within the fungal cell. This pattern suggests that secreted proteins with effector function may have evolved from non-secreted proteins without an effector function or vice versa. Second, changes in domain architecture of proteins within putative effector families also points to neo- or subfunctionalization. For example family 5485 contains MCO laccase-like enzymes and a single clade of three *Cqf* proteins that have acquired a MCO copper binding site in the evolution of this family. It is possible that these proteins have novel or distinct functions within *Cqf* than their paralogs within the genome. This family is a strong putative effector family because all *Cqf* members belong to the predicted secretome and it has undergone *Cqf*-specific family duplications. Protein members within this family co-localize in the genome, possibly resulting from tandem duplication from non-equal crossing over. Various functions have been ascribed to previously identified fungal MCOs including lignin degradation (Leonowicz et al., 2001; Lundell et al., 2010), melanin synthesis (Langfelder et al., 2003), fruiting body formation (Kues and Liu, 2000), and pathogenicity on hosts (Zhu and Williamson, 2004). This family has expanded in *Cqf,* the first sequenced rust pathogen that forms stem galls in woody tissues, and we hypothesize that these enzymes play a role in gall formation. The most common function for laccases/MCOs in basidiomycete fungi is lignin metabolism (Thurston, 1994; Kües and Rühl, 2011). However, this gene family exhibits a lack of conservation with known MCOs of lignin-degrading wood rots (*P. chrysosporium* and *S. lacrymans*), which points to the possibility that these enzymes may be involved in pathogenicity or may metabolize a plant substrate other than lignin. On both hosts, *Cqf* infects primary tissue that lacks high levels of lignification such as spongy mesophyll cells of oak leaves (Mims et al., 1996) and vascular cambium of pine (Gray et al., 1982). If the Family 5485 enzymes are involved in lignin degradation, the enzymatic activity may occur late in gall development on the pine host, where the tissues are more heavily lignified due to secondary wall formation. Though their biochemical targets are unknown *in planta*, we hypothesize that Family 5485 enzymes are secreted during infection and condition the gall phenotype on the pine host. Further studies are required to elucidate their true role in disease.

A second gene family that has expanded in the *Cqf* lineage is Family 9417, which includes five *Cqf*-specific paralogs that co-localize in the genome. Similar to Family 5485, differential domain architecture within this family implies that neo- or subfunctionalization may have occurred. Family 9417 contains putative effectors that harbor conserved, fungal-specific CFEM-domains. These domains exhibit a characteristic cysteine distribution and have a broad taxonomic conservation in fungi (Kulkarni et al., 2003; Martin et al., 2008; Perez et al., 2011). Predicted functions of proteins harboring CFEM domains include critical roles in appressorial development (Choi and Dean, 1997; DeZwaan et al., 1999), signal transducers, adhesion and cell-surface receptors (Kulkarni et al., 2003). In contrast to Family 5485 proteins, which may interact with the host during infection, the molecular target for Family 9417 proteins could be fungal. We hypothesize these proteins are secreted and may play roles during infection of the host.

### Evolution of gene gain and loss

Patterns of gene family loss and gain for rust fungi highlight major shifts in their proteomes, possibly associated with the rust pathogen's obligate biotrophic lifestyle. The origin of the rust pathogen clade is associated with nearly five times more losses, or family contractions, than duplications. There are many possible mechanisms for gene loss in rust fungi. For this reason, further investigations are required to both identify specific mechanisms and quantify their levels of effects on gene family evolution in rust fungi. However, we hypothesize that the since obligate biotrophy has evolved multiple times in fungi (Spanu, 2012), the skew toward gene loss in the rust pathogen lineage might be associated with the shift from the life history of its ancestral state to that of the obligate biotrophic pathogens we observe today. These lost and/or contracted families exhibit broad taxonomic conservation and may have been constituents of the ancestral “core” fungal gene set, suggesting that they are unnecessary for obligate biotrophic but may be necessary for free-living and symbiotic species. For example, enzymes integral to the sulfur and nitrogen assimilation pathways are missing in *Cqf* (unpublished, jgi.doe.gov/Cronartium), *Mlp*, and *Pgt* (Duplessis et al., 2011b). This also suggests that evolution for obligate biotrophy drives toward an irreversible life history shift (Spanu, 2012).

Although the rusts have undergone considerable gene family losses and contractions, they exhibit some of the largest proteomes in fungi. Much of their proteome size appears to be due to species-specific duplications. Nearly one-third (32.1%) of all observed duplications across all of the sampled basidiomycete fungi are rust taxon-specific duplications. The high levels of species-specific duplication yield disproportionately greater numbers of newly-evolved genes in the rust pathogen genomes compared to ancient or conserved genes (genes shared with older lineages) in each proteome. The presence of so many species-specific duplications suggests that the rusts have highly labile genomes. This is consistent with the large (>10%) genomic size variation detected in progeny from a single *Cqf* cross relative to parental isolates (Anderson et al., 2010). Such rapid changes, occurring in the span of a single generation, could facilitate the gene gains and losses observed in our analyses. The close association with hosts may foster a labile and diverse genome, enabling the parasites to rapidly adapt to the continually evolving host resistance pathways.

### Comparative analysis and genetic mapping to validate putative effectors

Further characterization of putative effectors in *Cqf* could be accomplished with analysis of selection potentially arising from host resistance mechanisms (Allen et al., 2004; Aguileta et al., 2009; Barrett et al., 2009; Thrall et al., 2012). In addition, expression analysis can be informative, since secreted proteins with specific expression profiles during infection are stronger effector candidates (Ellis et al., 2009). Time-course experiments have been successful in other rust pathogen systems to elucidate the effector-like proteins involved in multiple or highly specific stages during infection (Joly et al., 2010; Duplessis et al., 2011a; Bruce et al., 2014). Also, resequencing of closely related rust pathogens such as *Cronartium ribicola*, *C. flaccidum*, and *Peridermium harknessii* (Vogler and Bruns, 1998) would improve precision of gene family delineations and identification of true singleton *Cqf* effectors, which are likely to be more newly evolved than effectors in families, and may therefore be products of highly-specific host-*Cqf* coevolution. Finally, a subset of the predicted effectors are avirulence proteins and are, by definition, involved in genotype-specific “gene-for-gene” interactions with hosts. These putative avirulence effectors can be validated through genetic mapping to their corresponding host resistance genes, an approach that has previously been successful in identifying the first avirulence protein locus in *Cqf* (Kubisiak et al., 2011). Altogether, these validation approaches will yield true members of the *Cqf* secretome and provide additional insight into the biological functions for effectors infecting oak and pine.

### Conflict of interest statement

The authors declare that the research was conducted in the absence of any commercial or financial relationships that could be construed as a potential conflict of interest.
